# patGPCR: A Multitemplate Approach for Improving 3D Structure Prediction of Transmembrane Helices of G-Protein-Coupled Receptors

**DOI:** 10.1155/2013/486125

**Published:** 2013-03-11

**Authors:** Hongjie Wu, Qiang Lü, Lijun Quan, Peide Qian, Xiaoyan Xia

**Affiliations:** ^1^School of Computer Science and Technology, Soochow University, Suzhou 215006, China; ^2^Jiangsu Provincial Key Lab for Information Processing Technologies, Suzhou 215006, China; ^3^School of Electronic and Information Engineering, Suzhou University of Science and Technology, Suzhou 215009, China

## Abstract

The structures of the seven transmembrane helices of G-protein-coupled receptors are critically involved in many aspects of these receptors, such as receptor stability, ligand docking, and molecular function. Most of the previous multitemplate approaches have built a “super” template with very little merging of aligned fragments from different templates. Here, we present a parallelized multitemplate approach, patGPCR, to predict the 3D structures of transmembrane helices of G-protein-coupled receptors. patGPCR, which employs a bundle-packing related energy function that extends on the RosettaMem energy, parallelizes eight pipelines for transmembrane helix refinement and exchanges the optimized helix structures from multiple templates. We have investigated the performance of patGPCR on a test set containing eight determined G-protein-coupled receptors. The results indicate that patGPCR improves the TM RMSD of the predicted models by 33.64% on average against a single-template method. Compared with other homology approaches, the best models for five of the eight targets built by patGPCR had a lower TM RMSD than that obtained from SWISS-MODEL; patGPCR also showed lower average TM RMSD than single-template and multiple-template MODELLER.

## 1. Introduction

G-protein-coupled receptors (GPCRs) are among the most heavily investigated drug targets in the pharmaceutical industry [1] because activated GPCRs trigger a cascade of responses inside the cell. Although about 800 GPCRs in the human genome still await determining, the annual revenue in the market for human therapeutics based on the currently available receptors is in excess of $40 billion, and more than 50% of modern drugs are related to GPCRs [2]. On the other hand, it is still a very difficult problem to determine the conformation of GPCRs in vivo. The lipid environment in which the receptors are embedded blocks the two major techniques, nuclear magnetic resonance (NMR) spectroscopy and X-ray crystallography, that are used to determine protein structures. It is really exciting that the Nobel Prize in Chemistry was awarded in 2012 to two researchers studying the structure of GPCRs.

Fortunately, as demonstrated by recent publications [3, 4], in silico methods for deducing the three-dimensional structure of GPCRs have been increasingly successful. However, the development of computational approaches to predicting the structure of GPCRs remains a challenging task [5]. A lot of effort has been put into modeling the structures of the full-chain and the loop sections of GPCRs [6–8] and of membrane proteins [9, 10], whereas comparatively little research has been done on building more accurate models of the transmembrane helix sections of GPCRs, because the transmembrane helical bundles are commonly regarded as a conserved domain that can be easily duplicated from templates. In fact, the accuracy of the models of the transmembrane helix sections is still far away from the native structures, and it cannot meet the requirements for subsequent full-chain prediction and the modeling of ligand docking. For the GPCR Dock 2010 assessment [3] targets CXCR4/CVX15, CXCR4/IT1t, and D3, the averages of the TM RMSD values (the root mean square deviation of the backbone of the transmembrane helices) of all models submitted by the participants were 3.56 Å, 9.75 Å, and 2.55 Å, respectively, which are not high-resolution values (<2.0 Å). How can one build high-resolution models of the conformations of full-length GPCRs without an accurate transmembrane helical bundle? This paper addresses this problem using a parallelized multitemplate homology approach.

The classification of methods for the prediction of the three-dimensional structure of GPCRs in silico into homology modeling, threading, and ab initio folding follows the classification of methods for protein structure prediction. The homology method builds models starting from one or more template structures with a high sequence similarity. When the sequence identity between target and template is more than 50%, near-native models tend to be generated. When it is less than 30%, the accuracy of the models decreases sharply. SWISS-MODEL [11], Sybyl [12], Prime [13], MODELLER [14], NEST [15], SEGMOD/ENCAD [16], 3D-JIGSAW [17], and Builder [18] are widely used, stable, reliable, and accurate systems for homology modeling. The threading method operates by “threading” (i.e., placing and aligning) each amino acid (or amino acid segment) in the target sequence into a position in the three-dimensional structure and evaluating how well the target fits the template. Zhang et al. [6] used TASSER to generate structure predictions for all 907 putative GPCRs in the human genome. Based on a benchmarked confidence score, approximately 820 of the predicted models should have the correct folds. Ab initio prediction of protein structure involves modeling the dihedral angles for each residue based on the minimum-free-energy principle, without the use of any experimentally solved structures. Baker's group [19, 20] was successful in the recent CASP (the Critical Assessment of protein Structure Prediction) challenge and designed a membrane-environment-related energy function to guide membrane protein folding.

Homology approaches can be divided into two categories, namely, single-template and multiple-template, depending on the number of templates employed in modeling. Single-template homology approaches cannot always achieve the best results, owing to difficulties in detecting the best template, particularly when remote homology is detected [21]. Multitemplate approaches can effectively combine more reasonably aligned regions than the single-template approach Cheng [22] reported that a multitemplate combination algorithm improved the GDT-TS scores of the predicted models by 6.8% on average for 45 CASP7 comparative-modeling targets. Liu et al. [23] took into account the information represented by multiple templates and alignments at the three-dimensional level by mixing and matching regions between different initial comparative models, and the multitemplate approach produced conformational models of higher quality than the individual starting predictions. MODELLER [14] modeled 3D conformations by optimally satisfying spatial restraints derived from the alignment and expressed as probability density functions for the features restrained. NEST [15] produced a model by taking a sequence alignment of a target to one or multiple template PDB files as input. 3D-JIGSAW [17] used a convergence of algorithms for comparative modeling which led to more reliable structures by superimposing multiple known structures from a protein family. In our opinion, previous multitemplate approaches have generally built a “super” template, which might ignore the flexibility of aligned structures from different templates. The long-distance homology information from different templates should be exchanged in each iteration rather than directive merging structures.

This paper proposes a parallelized multitemplate approach, inspired by our previous method pacBackbone [24, 25], for the prediction of the three-dimensional structure of the transmembrane helices of GPCRs. The system proposed here is referred to as “patGPCR” (parallelized multitemplate approach to GPCR transmembrane helix structure prediction). Parallelization not only accelerates the running speed but also provides a novel and effective mechanism to exchange homology regions between templates softly. We have exploited our method to predict tertiary structures for all eight determined GPCRs published on the GPCR Network website (http://gpcr.scripps.edu/index.html). Compared with other homology approaches, the best models for five of the eight targets built by patGPCR had a lower TM RMSD than was obtained from SWISS-MODEL, patGPCR showed lower average TM RMSD than single-template MODELLER, and patGPCR showed only one higher average TM RMSD target compared with multiple-template MODELLER. 

## 2. Materials and Methods

### 2.1. Parallelized Framework of patGPCR

**Figure 1:**
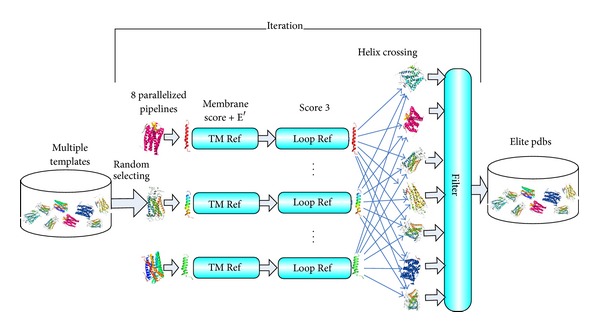
Parallelized framework of patGPCR.

 GPCRs share a similar structural topology, composed of seven transmembrane (TM) helices packed into a 7-TM helical bundle, with three intracellular (icls) and three extracellular loops (ecls) [26]. Thus, single helical refinement should be paralleled in independent threads. The parallelized framework for patGPCR is depicted in Figure 1. At the beginning of patGPCR, top 2–4 templates were examined for sequence identities using the Protein Data Bank (http://www.rcsb.org/pdb), which were used as starting template conformations. Eight parallelized pipelines which involve independent subprocedures (or threads) were used to randomly select starting conformations of the templates. Each pipeline containing TM refinement and loop refinement optimized an adjacent helix pair region. The first and the last pipelines optimized only one helix and one terminus. To use reasonable structural regions within different templates, TM helix crossing step and elite pool were introduced into the parallelized framework at the end of the pipelines. In the crossing step, each pipeline shared the best-so-far helix with other pipelines. If lower energy was obtained by helix crossing, the helix substitution was accepted; otherwise, it was rejected. Optimized conformations are conserved in the elite pool prepared for the next iteration. Thus, the crossing step and elite pool are two critical mechanisms of patGPCR to identify reasonable structures from multiple templates to pipelines and iterations.

### 2.2. Multiple Templates for Eight GPCR Targets

 patGPCR was evaluated by using blind prediction testing the set of the eight determined GPCRs published on the GPCR network. Amino acid sequence was the only input used for blind prediction, which is commonly employed in GPCRDock2008/2010 and CASP exercises. This is the largest set used for directly evaluating prediction results. patGPCR employed 2–4 templates (column 4 in Table 1) for each target in the test set. The top three or four templates were selected by standard protein BLAST based on default parameters. Most sequence similarities between the templates and targets were lower than 50% and average sequence similarity for the templates was 36% (column 6 in Table 1). In some cases, the templates have high-sequence identity. There are two reasons why we did not remove these cases from benchmark. First, we are interested in validating the modeling ability of patGPCR based on various templates with different sequence identities. Second, patGPCR, SWISS-MODEL, and MODELLER used the same templates in the comparing experiments, so the results we presented are fair for these methods, no matter high- or low-sequence identity the templates is. 

### 2.3. TM Refinement Protocol

 The 7-TM helical bundle is the primary topology of GPCRs, which comprises approximately 75% of amino acids in the entire protein chain. The TM helix has been conserved throughout evolution [5]. However, in a recent GPCRDock2008/GPCRDock2010 study, average TM RMSDs of CXCR4/CVX15, CXCR4/IT1t, and D3 were 3.56 Å, 9.75 Å, and 2.55 Å. The accuracy of predicting TM regions can be improved, and the correct TM position and orientation ensure that loop regions are properly oriented. TM refinement protocols employed using patGPCR are based on 7-TM geometrical topology reflecting the bundle structure using a set of geometrical parameters. Topologically, each TM helix is regarded as a rigid body, and relative positions of internal atoms remain fixed when moving or rotating the rigid body.

TM refinement is divided into four stages. In the first stage, patGPCR was used to identify TM boundaries by averaging the results of six existing methods: TopPred [27], UniProt [28], TMpred [29], HMMTOP [30], TMHMM [31], and OCTOPUS [32]. In the second stage, translation of each rigid body along, or perpendicular to, the axis of the helix was used to optimize the relative positions of seven helices. In the third and fourth stages, spin angels and tilt angles were refined, respectively. 

### 2.4. Loop Refinement Protocol

 patGPCR, which involves an entirely predictive approach for GPCRs, combines two Rosetta loop modeling protocols. Due to high flexibility in loop regions, ab initio methods typically involve calculation of possible loop conformations with the help of various energy functions and minimizations [33–35]. patGPCR paralleled two Rosetta loop modeling protocols, including CCD [36] and KIC [37] modeling with Rosetta energy function *score*3. Each pipeline was used to randomly choose a loop movement to refine the loop section. 

### 2.5. The Algorithm Executed by Single Pipeline

Algorithm 1 executed by single pipeline contains TM and loop refinement at lines 2–15 and line 17, respectively. In the 2nd line, function *getHelixAxis*() gets the direction vector of the axis of the helix $\overset{\to }{d_{TM}}[]$. In the 3rd line, function *getMembrane*() gets the normal vector of the membrane plane $\overset{\to }{d_{M}}$. The 4th–7th lines execute second TM refinement stage and function *transferHelix*(*P*, *TM*[], *RND*(−5,5), *RND*(−5,5), *RND*(−5,5)) randomly translates the helix from current position along *x*-, *y*-, or *z*-axis ranging from −5 Å to 5 Å. The new helix position would be accepted if the new energy *E*_*m*_ is lower than the energy before translating. The 8th–11th lines execute the third TM refinement stage and function $spinHelix(P,TM[],\overset{\to }{d_{TM}}[],i)$ spins the helix from −180 to 180 degrees and validates the new position using energy function *E*_*m*_. The 12th–15th lines execute the fourth TM refinement stage and function *tiltGaussianHelix* $\left(P,TM[],\overset{\to }{d_{M}},30,5\right)$ samples the tilt angles between the helix and the normal plane of transmembrane according to gaussian distribution (expected value is 30 and the variance is 6). In the 16th line, function *LoopModelRandomly*() refines the loop regions using Rosetta KIC or CCD remodel protocol randomly. In the 17th line, function *crossHelices*() exchanges the helixes among pipelines. 

### 2.6. Energy Item for Evaluating the Helical Bundles

**Figure 2:**
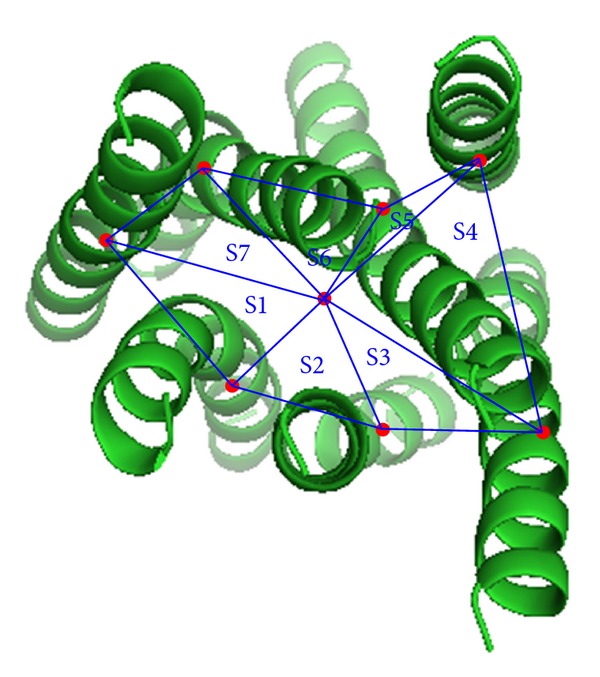
Parallelized framework of patGPCR.

 Developing an appropriate energy function remains a challenge in predicting GPCR 3D structures. An accurate energy function can be used to distinguish near-native models from candidates, while an imprecise energy function may not recognize near-native models even if they have been sampled by using an accurate search algorithm. patGPCR improves the Rosetta membrane energy function [19] by including a novel energy item *E*′ for evaluating rigid helix packing. After projecting helices onto the membrane plane, Figure 2 was constructed to show *S*1–*S*7, which is the area of triangles constituted by the center point *O* and two intersection points of adjacent helix axes with the membrane plane. In (1), *S* is the sum of *S*1–*S*7 and *S*_min⁡_ and *S*_max⁡_ are the maximum and minimum values of *S* in known structures of GPCRs. A smaller *E*′ indicates a tighter 7-TM helical bundle, while a greater *E*′ indicates a looser bundle. Detection of collisions between residues is included in the Rosetta membrane energy function. Thus, the Rosetta membrane energy function which includes an additional item *E*′ is employed by the patGPCR TM refinement algorithm:

(1) E′={(S−Smin⁡)2Smin⁡2,S<Smin⁡,0,Smin⁡<S<Smax⁡,(S−Smax⁡)2Smax⁡2,S<Smax⁡,

## 3. Results

### 3.1. The Contribution of TM Refinement Protocol Employed by Single Pipeline

 The contributions of TM refinement protocols were investigated on the comparison with models submitted by GPCRDOCK2010 participants. GPCRDOCK2010 exercise is a community-wide assessment for GPCR homology-modeling and docking. The participants submitted the best five candidates of targets CXCR4 and D3. We executed patGPCR to optimize the helical bundles starting from the submitted conformations downloaded from GPCRDOCK2010 official website. The TM RMSD of the best model after refining and top 5 models published on GPCRDOCK2010 are listed in Table 2. For CXCR4 (D3) target, the TM RMSD of the best model after refining is 2.165 Å (0.93 Å), which ranks 4th (1st) among 161 (168) submitted models. 

### 3.2. The Contribution of Parallelized Multitemplate

 We examined the contributions of multiple-templates versus a single-template of patGPCR with the same settings. The number of templates used by the patGPCR ranged from two to four. Average of sequence identities between the templates and targets was 36%. Compared to multitemplate patGPCR, single-template patGPCR employed only one template and did not utilize the helix crossing step.

**Figure 3:**
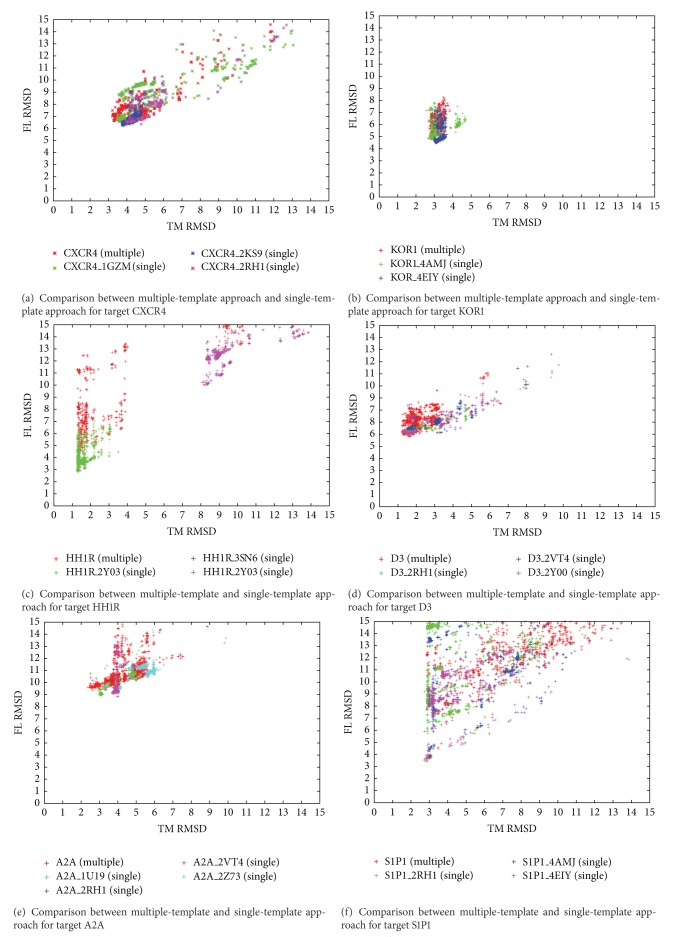

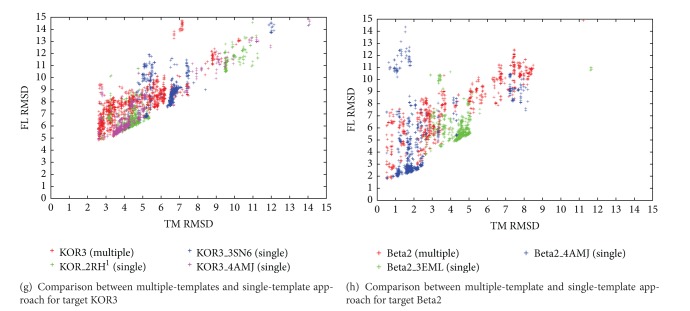
Comparison of multiple-template and single-template patGPCR for eight GPCR targets. The *x*- and *y*-axes indicate TM RMSD and FL RMSD, respectively. Results generated using the multitemplate approach are shown in red and those generated using the single-template version of patGPCR are shown in green, blue, and purple. For five out of eight targets, CXCR4 (a), D3 (d), A2A (e), KOR3 (g), and Beta2 (h), the multitemplate approach yielded a higher number of conformations with lower TM RMSD values than the single-template approach. For KOR1 (b), the narrow sampling space in any single template may have resulted in similar performances for the multiple-template and single-template approaches.

Full-length RMSD (FL RMSD) of GPCR and TM RMSD comparisons are plotted in Figure 3; dots with higher RMSD values (>15 Å) are not included. The average improvement in TM RMSD using the multiple-template patGPCR was 33.64% (raw TM RMSD increase 1.57 Å, columns 4 and 8 in Table 1).

Multiple-template patGPCR yielded a higher number of low TM RMSD conformations than the single-template patGPCR in most cases (five out of eight targets), including CXCR4 (Figure 3(a)), D3 (Figure 3(d)), A2A (Figure 3(e)), KOR3 (Figure 3(g)), and Beta2 (Figure 3(h)). In other cases, the multitemplate approach showed similar performance with single-template patGPCR, including KOR1, HH1R, and S1P1 in Figures 3(b), 3(c), and 3(f). For KOR1, the narrow sampling space in the single template may have resulted from similar performance between multiple-template and single-template patGPCR. For HH1R and S1P1, both multiple-template and single-template patGPCR appeared to show bottlenecks. One reason for this may be that the transmembrane refining protocol employed by multiple-template and single-template approaches failed in some cases. Further analysis is described in Section 4.3. 

### 3.3. Comparison of patGPCR to Homology Approach SWISS-MODEL

 The SWISS-MODEL [16] is a widely used homology modeling approach which provides online prediction and manual template specification. We executed the SWISS-MODEL to predict eight targets using the same templates as used in patGPCR (Table 1). Three predictions that failed using the SWISS-MODEL are indicated as “×” in column 9 in Table 1.

**Figure 4:**
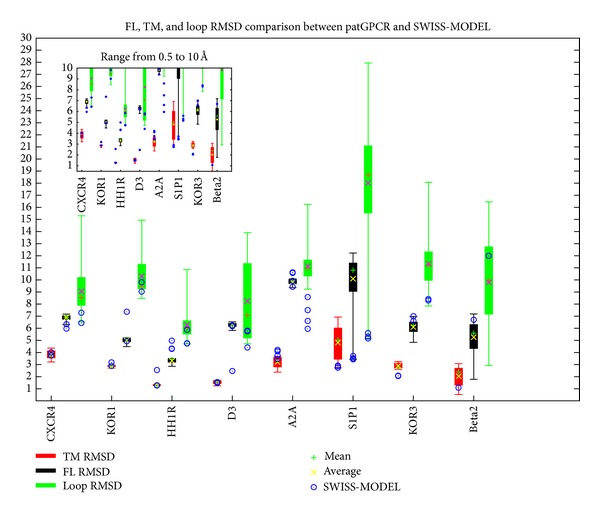
A box-and-whisker plot comparing FL, TM, and Loop RMSD between patGPCR and the SWISS-MODEL. The maximum, minimum, 1st quartile, 3rd quartile, mean (shown by +), and average (shown by ×) of RMSD (*y*-axis in angstrom) of FL, TM, and loop RMSD values are shown in red, black, and green for each target (*x*-axis). The results of the SWISS-MODEL are marked as blue circles in the corresponding box. The left-top panel is a detailed illustration in the range from 0.5 Å to 10 Å.

**Figure 5:**
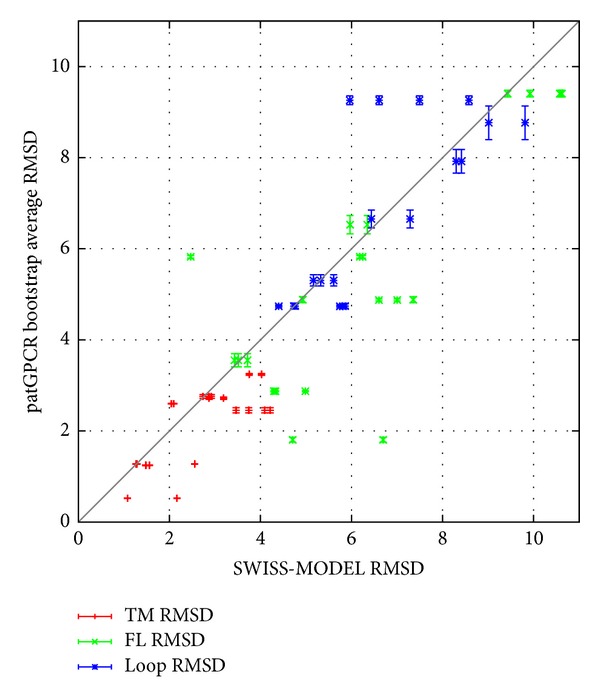
Statistical comparison between patGPCR after a 50-fold bootstrap estimation and the SWISS-MODEL on FL, TM, and Loop RMSD. *x*-axis indicates 1-percentile average RMSD of 50-fold bootstrap estimation of patGPCR prediction results and *y*-axis indicates the RMSD of the SWISS-MODEL. The error bars of the points indicate standard deviation 1 percentiles of patGPCR. Red, green, and blue show the comparison on TM, FL, and loop RMSD values, respectively. Points below the line represent targets where patGPCR yields higher accuracy, and above the line the SWISS-MODEL yields higher accuracy.

For each decoy (set of conformations of the predicted result) predicted by patGPCR, the top 400 conformations in terms of TM RMSD were reserved, while the others were eliminated from the decoy. Thus, we simplified selection of the nearest native prediction from the decoys. We depicted the comparison of decoys from the SWISS-MODEL results using a box-and-whisker plot (Figure 4). Prediction accuracy was expressed as the RMSD in angstroms, which was calculated after superimposing the corresponding coordinates of alpha-carbons determined by prediction and those of the native structure. To accurately determine transmembrane helices, the best model of five targets (CXCR4, KOR1, D3, A2A, and Beta2) yielded by patGPCR showed lower TM RMSD values than those determined using the SWISS-MODEL. patGPCR showed similar transmembrane accuracy on two targets (HH1R and S1P1); another target (KOR3), patGPCR, was inferior to the target identified using the SWISS-MODEL. In Figure 4, the accuracy of full-length chain and loop regions is shown. For four of the eight targets (KOR1, HH1R, KOR3, and Beta2), the best models predicted by patGPCR showed lower full-length RMSD values than those generated using the SWISS-MODEL. It is not surprising that only three targets (KOR1, KOR3, and Beta2) for patGPCR were more accurately predicted compared to the SWISS-MODEL regarding the accuracy of loop regions since patGPCR places emphasis on helix optimization, whereas relatively little emphasis is placed on loop optimization and the loop regions were excluded in the crossing step. Next, statistical properties of the representative solutions, the top 400 conformations regarding TM RMSD, for each decoy set were investigated. For each result decoy, Figure 5 shows the 1-percentile average (*x*-axis) and standard deviation (error bars of symbols) for every test instance, calculated from 50-fold bootstrap estimation using the BioShell package [38]. We choose the 1 percentile compared with the SWISS-MODEL results (*y*-axis) since only some of the decoys will be retained for further analysis, such as side chain refinement, ligand docking, and virtual screen. In Figure 5, patGPCR showed 14, 16, and 11 better results (below the line) for a total of 20 symbols on TM, FL, and loop RMSD values, respectively. In Figure 5, the lower deviations of the error bars indicate that patGPCR exhibits robust performance in most cases. 

### 3.4. Comparison of patGPCR to Homology Approach MODELLER

 MODELLER [14] is a well-known tool used for single-template and multiple-template homologies or comparative modeling of protein three-dimensional structures. We conducted single-template and multiple-template experiments using MODELLER on the same Linux system and hardware as was used for patGPCR. For a reasonable comparison, single-template MODELLER generated 1300 predicted conformations, which was similar to the average predicted number of 1358.75 conformations generated by patGPCR, for each target and each template. Multiple-template MODELLER was also used to generate 1300 prediction conformations for each target. MODELLER also employed the same templates as patGPCR listed in Table 1. MODELLER aligning commands, including align2d() and salign(), which reportedly take into account structural information from the template when constructing an alignment, were used to align templates to the targets.

MODELLER experimental results are tabulated in columns 10 and 11 in Table 1. The 10th column shows the average and standard deviation of TM RMSD of conformations generated using the single-template MODELLER, and the 11th column shows the average and standard deviation of TM RMSD of conformations generated by the multiple-template MODELLER. For six out of eight targets, patGPCR showed lower average TM RMSD than single-template MODELLER, and patGPCR showed only one higher average TM RMSD target compared with multiple-template MODELLER. Multiple-template patGPCR yielded a larger number of lower TM RMSD conformations than the single-template patGPCR for most cases (five out of eight targets), including CXCR4, D3, A2A, KOR3, and Beta2. However, no target was improved by using multiple-template MODELLER. The average TM RMSD values for conformations generated by using multiple-template MODELLER were nearly two times higher than those generated using single-template MODELLER. The lower standard deviation in column 11 indicates that a narrow structural sampling strategy was adopted by multiple-template MODELLER, which may have led to the multiple-template MODELLER being trapped by an unaligned region.

**Figure 6:**
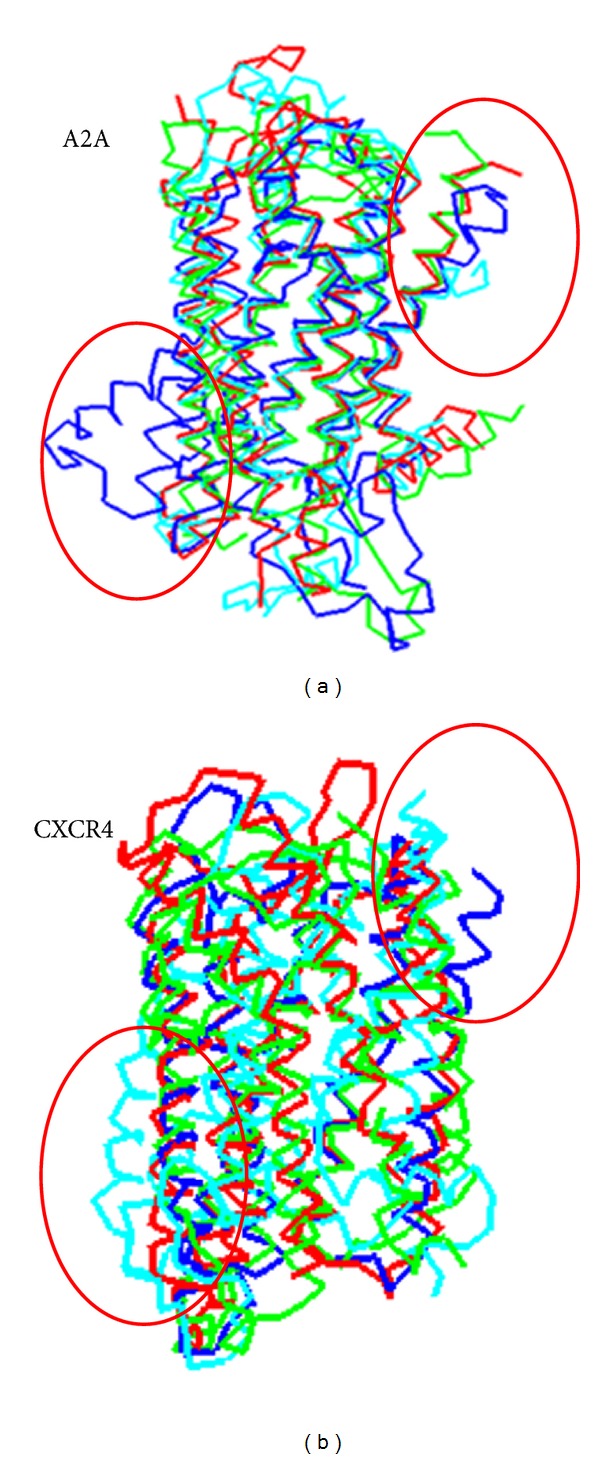
Superimposition of conformations yielded by native (red), patGPCR (green), SWISS-MODEL (blue), and MODELLER (cyan). The four red cycles indicate that patGPCR (green) backbones in the TM region are closer to native (red) than those predicted using the SWISS-MODEL (blue) and MODELLER (cyan) in targets A2A (a) and CXCR4 (b).

Finally, a direct visual comparison of conformations yielded by native, patGPCR, SWISS-MODEL, and MODELLER are depicted in Figure 6. 

## 4. Discussions

### 4.1. Complexity Analysis of patGPCR

 Since patGPCR does not synchronize the exchange of helices between pipelines, the complexity of patGPCR depends on that of a single pipeline repeating Algorithm 1 *T* times. For a GPCR target with *n* amino acid residues, the functions *transferHelix*(), *spinHelix*(), and *tiltGaussianHelix*() execute a movement of each residue in the helices, so these three functions have the same time complexity, *O*(*n*). Therefore, the complexity of the TM refinement contained in the three stages from the fourth to the 15th line of Algorithm 1 is 3 × *O*(*n* ^2^). The function *crossHelices*() exchanges the residues in each pair of helices and has complexity *O*(*n* ^2^). The complexity of *LoopModelRandomly*() is determined by that of the Rosetta CCD and KIC refinement protocols, which are both composed of two-layer iterations repeating *outer*_*cycles* and *inner*_*cycles* (Rosetta parameters). Moreover, we generally set the parameters *T*, *STAGE*2_*CYCLES* (in the fourth line of Algorithm 1), *STAGE*4_*CYCLES* (in the 12th line of Algorithm 1), *outer*_*cycles*, and *inner*_*cycles* in a way that is linearly dependent on *n*. In summary, the time complexity of the parallelized patGPCR is eight times of that of a single pipeline, which is *n* × (3 × *O*(*n* ^2^) + *O*(*n* ^2^) + *O*(*n* ^2^)) = *O*(*n* ^3^).

The space complexity of patGPCR is mainly determined by the elite conformation pool shown in Figure 1, the set of residue numbers for the transmembrane regions *TM*[], and the set of residue numbers for the loop regions *Loop*[]. The pool is designed to store the coordinates of four backbone atoms of the elite conformations with the same size of template, so it requires 4 × 3 × *n* × *F* space, where *F* is the number of available templates for the corresponding GPCR targets. *TM*[] and *Loop*[] obviously require *O*(*n*) space. The space complexity of patGPCR is then *O*(4 × 3 × *n* × *F*) + 2 × *O*(*n*) = *O*(*n*). 

### 4.2. Why Does the patGPCR Approach Work in General?

 The following factors may contribute to the improvement in transmembrane helix structural prediction. First, a multitemplate approach can effectively combine more reasonably aligned regions than a single-template approach. Single-template approaches use the top-ranked template and its alignment with the target protein to model the structure. These approaches cannot always achieve the best results, owing to difficulties in detecting the best template, particularly when remote homology is detected between targets and templates [39]. Because most of the sequence similarities among GPCRs are less than 50%, it is unreliable to attempt to obtain high-resolution models using only a single template. We believe that each template, even if it has lower sequence similarity (<30%), contains a reasonable structure for the targets overall. Multiple-template approaches can not only increase the alignment coverage but also broaden the conformation search space by extracting aligned fragments from different templates.

Second, the parallelized approach enables an effective schema for exchanging reasonable regions among multiple templates. Previous multitemplate approaches [22, 40] have generally built a “super” template by methods that involve very little merging of aligned fragments from different templates, whereas the parallelized schema of patGPCR includes a soft resolution for introducing aligned regions and exchanges the reasonable helices between different pipelines at the end of an iteration. Therefore, the contribution of aligned regions from different templates will not only influence the beginning of the algorithm but also be preserved in subsequent iterations. Fortunately, because the helices that the GPCRs have in common are conserved, the transmembrane refinement protocol (see Section 2.3) will improve the accuracy of the aligned regions by moving the coordinates of the atoms toward lower free energy. This also helps the parallelized schema to broadcast the reasonable regions to the elite conformation pool.

Third, the additional energy item *E* related to the helical bundles (see Section 2.6) can reasonably guide the helices toward assembling into a tighter bundle, although not perfectly. The combination of the Rosetta membrane score with *E* based on the GPCR targets is effective in patGPCR, as shown in our experiments.

### 4.3. Limitations of patGPCR

 On the other hand, patGPCR can also be improved in some respects. First, owing to the inadequately known structures of GPCRs, we could only validate the strengths and weaknesses of patGPCR on eight determined cases published on the GPCR Network website. These eight targets are the largest test data set for GPCRs at present. Therefore, the question of whether the current results of our experiments are occasional instances or have some universality cannot be answered immediately. Second, patGPCR seemingly encountered a bottleneck in some cases (in Figures 3(c) and 3(f)); it was difficult for patGPCR to obtain an accuracy results than single-template approach. The bottleneck may have two reasons: (a) the energy term in (1) improves the Rosetta energy function, but it is still not good enough to distinguish between the near-native conformations and (b) in patGPCR, the helix is treated as a rigid body, unlike real helices, which have disorder, irregular regions, and flexibility. The coordinates of the atoms in the interior of the helix are likely to cause a loss of precision. Third, although patGPCR generates a higher quality of decoys in the transmembrane regions, the difficulty of building accurate models of loop regions with high flexibility is a consequence of the difficult issues of the GPCR structure prediction problem.

### 4.4. Ability of New Energy Item

 The ability of the new energy item *E*′ was investigated for six GPCR targets, including CXCR4, KOR1, D3, A2A, KOR3, and Beta2, whose average TM RMSDs were lower than 5 Å. The relatively lower average TM RMSD values gained by using both patGPCR and MODELLER indicate that the accuracy of these targets depend on the energy functions. In Table 3, the top quarter of conformations in terms of TM RMSD values from the decoy of multiple-template patGPCR were chosen to compare how many conformations could be predicted using MEM (Rosetta membrane score) and *E*′ (new energy item). For four targets (KOR1, D3, A2A, and KOR3), *E*′ identified more nearly native conformations than MEM. Only one target (CXCR4) was identified to have fewer conformations than MEM. 

## 5. Conclusion

 In this paper, we have presented a parallelized multitemplate approach, patGPCR, to predicting the 3D structure of the transmembrane helices of G-protein-coupled receptors. patGPCR, which employs a bundle-packing-related energy function that improves on the RosettaMem energy, parallelizes eight pipelines for the refinement of transmembrane helix structures and exchanges the optimized helix structures among multiple templates. We have investigated the performance of patGPCR on a test set containing eight determined GPCRs published on the GPCR Network website. The results indicate that our multitemplate algorithm improves the TM RMSD values of the predicted models by 33.64% on average against a single-template method. Compared with other ab initio and homology approaches, patGPCR yielded more predicted conformations with high-resolution structures of the transmembrane helices. The best models for five of the eight targets built by patGPCR had a lower TM RMSD than had the models obtained from SWISS-MODEL, and half of them had a lower full-length RMSD. For six out of eight targets, patGPCR showed lower average TM RMSD than single-template MODELLER, and patGPCR showed only one higher average TM RMSD target compared with multiple-template MODELLER.

## Supplementary Material

The data sets include the conformations files generated by single template and multi-template patGPCR and the results files from SWISS-MODEL Online.
The file patGPCR.rar is the results of patGPCR method.
The file Swiss.rar is the results of Swiss method.
The file Modeller.rar is the results of Modeller method.

## Figures and Tables

**Algorithm 1:**
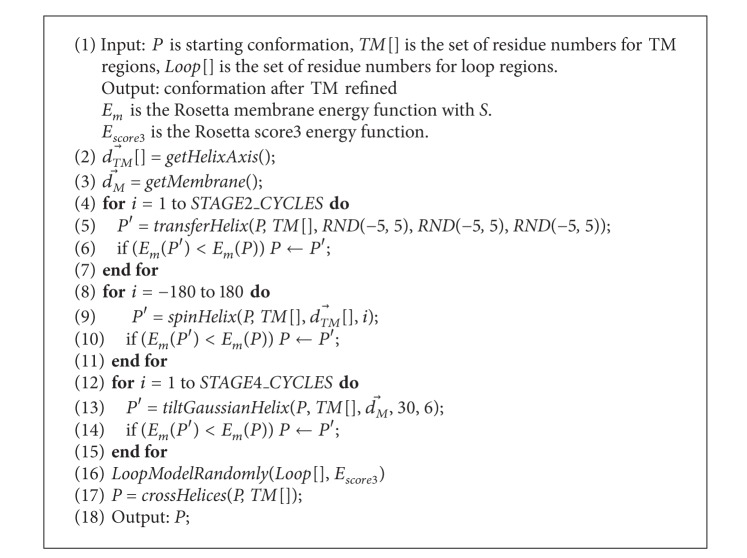
Refinement by single pipeline (P, *TM* [], *Loop* []).

**Table 1:** Eight determined GPCR targets and their templates.

Target names	Full names	No. of MT pat^a^	Templates	Chain	SS^b^	Avg ST pat^c^	Avg MT pat^d^	SWISS^e^	Avg ST MOD^f^	Avg MT MOD^g^
			1GZM	A	24%	6.34		*✓*		
CXCR4	CXCR4	932	2KS9	A	30%	4.33	4.26 ± 1.40	*✓*	7.69	11.4 ± 0.16
			2RH1	A	31%	5.77		×		

KOR1	kappa opioid receptor	1218	4AMJ	A	27%	3.23	2.98 ± 0.22	*✓*	3.37	7.11 ± 0.20
			4EIY	A	56%	3.40		*✓*		

			2Y03	A	36%	1.56		*✓*		
HH1R	Histamine receptor H1	1533	3SN6	R	47%	20.64	12.17 ± 10.65	*✓*	3.87	17.32 ± 0.24
			4AMJ	A	33%	11.17		*✓*		

			2RH1	A	44%	2.64		*✓*		
D3	D3 dopamine receptor	1776	2Y00	A	34%	3.40	1.69 ± 0.81	*✓*	1.75	2.61 ± 0.16
			2VT4	A	36%	3.20		*✓*		

			1U19	A	31%	4.35		*✓*		
A2A	adenosine A2a receptor	2076	2RH1	A	44%	26.58	3.53 ± 21.52	*✓*	3.85	7.88 ± 0.30
			2VT4	A	35%	4.16		*✓*		
			2Z73	A	23%	5.80		*✓*		

			2RH1	A	27%	4.39		*✓*		
S1P1	Sphingosine 1-phosphate receptor 1	1031	4EIY	A	54%	5.43	6.67 ± 2.69	*✓*	10.83	23.71 ± 0.77
			4AMJ	A	29%	5.52		*✓*		

			2RH1	A	29%	5.00		*✓*		
KOR3	nociceptin opioid receptor	1409	3SN6	R	35%	7.73	3.41 ± 1.67	×	8.71	16.82 ± 0.43
			4AMJ	A	37%	4.29		*✓*		

Beta2	beta-2-adrener gic receptor	895	3EML	A	33%	5.60	3.82 ± 2.60	×	1.36	1.54 ± 0.28
			4AMJ	A	64%	2.19		*✓*		

Avg	1358.75				36%	6.38	4.81 ± 5.20		5.11	11.05 ± 0.32

^a^Number of conformations generated by MT (multiple-template) patGPCR.

^b^Sequence similarity.

^c^Average TM RMSD of conformations generated by ST (single-template) patGPCR.

^d^Average TM RMSD of conformations generated by MT (multiple-template) patGPCR.

^e^SWISS output.

^f^Average TM RMSD of conformations generated by ST (single-template) MODELLER.

^g^Average TM RMSD of conformations generated by MT (multiple-template) MODELLER.

**Table 2:** Comparison with GPCRDocking2010 participants.

Number	Group name	CXCR4 TM RMSD	Group name	D3 TM RMSD
1	UMich-Pogozheva	2.05	UMich-Zhang	1.26
2	UMich-Zhang	2.08	Monash-Hall	1.35
3	VU-MedChem	2.14	WUStL	1.35
4	Monash-Sexton-1	2.18	Monash-Sexton-2	1.37
5	UMich-Zhang	2.18	WUStL	1.37
6	patGPCR	2.165	patGPCR	0.930

**Table 3:** Comparison of MEM and *E*′.

Target names	Number^a^	Neither^b^	Only *E*′^c^	Only MEM^d^	Both^e^
CXCR4	232	137	8	87	0
KOR1	304	107	86	52	59
D3	444	249	82	75	38
A2A	520	72	192	133	123
KOR3	352	172	84	74	22
Beta2	224	77	42	42	63

^a^The number of top quarter conformations in terms of TM RMSD from the decoy.

^b^The number of conformations that was neither in top quarter conformations in terms of MEM nor in top quarter conformations in terms of *E*′.

^c^The number of conformations that was only in top quarter conformations in terms of *E*′.

^d^The number of conformations that was only in top quarter conformations in terms of MEM.

^e^The number of conformations that was both in top quarter conformations in terms of MEM and in top quarter conformations in terms of *E*′.

## References

[1] Fillmore D (2004). It’s a GPCR world. *Modern Drug Discovery*.

[2] Cherezov V, Rosenbaum DM, Hanson MA (2007). High-resolution crystal structure of an engineered human *β*2-adrenergic G protein-coupled receptor. *Science*.

[3] Kufareva I, Rueda M, Katritch V (2011). Status of GPCR modeling and docking as reflected by community-wide GPCR Dock 2010 assessment. *Structure*.

[4] Michino M, Chen J, Stevens RC, Brooks CL (2010). FoldGPCR: structure prediction protocol for the transmembrane domain of G proteincoupled receptors from class A. *Proteins*.

[5] Katritch V, Rueda M, Lam PCH, Yeager M, Abagyan R (2010). GPCR 3D homology models for ligand screening: lessons learned from blind predictions of adenosine A2a receptor complex. *Proteins*.

[6] Zhang Y, Devries ME, Skolnick J (2006). Structure modeling of all identified G protein-coupled receptors in the human genome. *PLoS Computational Biology*.

[7] de Graaf C, Foata N, Engkvist O, Rognan D (2008). Molecular modeling of the second extracellular loop of G-protein coupled receptors and its implication on structure-based virtual screening. *Proteins*.

[8] Hildebrand PW, Goede A, Bauer RA (2009). SuperLooper—a prediction server for the modeling of loops in globular and membrane proteins. *Nucleic Acids Research*.

[9] Sun L, Zeng X, Yan C (2012). Crystal structure of a bacterial homologue of glucose transporters GLUT1-4. *Nature*.

[10] Hopf TA, Colwell LJ, Sheridan R (2012). Three-dimensional structures of membrane proteins from genomic sequencing. *Cell*.

[11] Bordoli L, Kiefer F, Arnold K, Benkert P, Battey J, Schwede T (2009). Protein structure homology modeling using SWISS-MODEL workspace. *Nature Protocols*.

[12] (2009). *Sybyl-X, Version 1. 0*.

[13] (2007). *Prime, Version 1.6*.

[14] Sali A, Blundell TL (1993). Comparative protein modelling by satisfaction of spatial restraints. *Journal of Molecular Biology*.

[15] Petrey D, Xiang Z, Tang CL (2003). Using multiple structure alignments, fast model building, and energetic analysis in fold recognition and homology modeling. *Proteins*.

[16] Levitt M (1992). Accurate modeling of protein conformation by automatic segment matching. *Journal of Molecular Biology*.

[17] Bates PA, Kelley LA, MacCallum RM, Sternberg MJE (2001). Enhancement of protein modeling by human intervention in applying the automatic programs 3D-JIGSAW and 3D-PSSM. *Proteins*.

[18] Koehl P, Delarue M (1994). Application of a self-consistent mean field theory to predict protein side-chains conformation and estimate their conformational entropy. *Journal of Molecular Biology*.

[19] Yarov-Yarovoy V, Schonbrun J, Baker D (2006). Multipass membrane protein structure prediction using Rosetta. *Proteins*.

[20] Barth P, Schonbrun J, Baker D (2007). Toward high-resolution prediction and design of transmembrane helical protein structures. *Proceedings of the National Academy of Sciences of the United States of America*.

[21] Cavasotto CN, Phatak SS (2009). Homology modeling in drug discovery: current trends and applications. *Drug Discovery Today*.

[22] Cheng J (2008). A multi-template combination algorithm for protein comparative modeling. *BMC Structural Biology*.

[23] Liu T, Guerquin M, Samudrala R (2008). Improving the accuracy of template-based predictions by mixing and matching between initial models. *BMC Structural Biology*.

[24] Lü Q, Xia X, Chen R (2012). When the lowest energy does not induce native structures: parallel minimization of multi-energy values by hybridizing searching intelligences. *PLoS ONE*.

[25] Lü Q, Wu H, Wu J, Huang X, Luo X, Qian P A parallel ant colonies approach to de novo prediction of protein backbone in CASP8/9.

[26] Lefkowitz RJ (2000). The superfamily of heptahelical receptors. *Nature Cell Biology*.

[27] Claros MG, von Heijne G (1994). TopPred II: an improved software for membrane protein structure predictions. *Computer Applications in the Biosciences*.

[28] uniprot http://www.uniprot.org/.

[29] Hofmann K, Stoffel W (1993). Tmbase—a database of membrane spanning proteins segments. *Biological Chemistry Hoppe-Seyler*.

[30] Tusnády GE, Simon I (2001). The HMMTOP transmembrane topology prediction server. *Bioinformatics*.

[31] Krogh A, Larsson B, von Heijne G, Sonnhammer ELL (2001). Predicting transmembrane protein topology with a hidden Markov model: application to complete genomes. *Journal of Molecular Biology*.

[32] Viklund H, Elofsson A (2008). OCTOPUS: improving topology prediction by two-track ANN-based preference scores and an extended topological grammar. *Bioinformatics*.

[33] Soto CS, Fasnacht M, Zhu J, Forrest L, Honig B (2008). Loop modeling: sampling, filtering, and scoring. *Proteins*.

[34] Rapp CS, Strauss T, Nederveen A, Fuentes G (2007). Prediction of protein loop geometries in solution. *Proteins*.

[35] Zhu K, Pincus DL, Zhao S, Friesner RA (2006). Long loop prediction using the protein local optimization program. *Proteins*.

[36] Mandell DJ, Coutsias EA, Kortemme T (2009). Sub-angstrom accuracy in protein loop reconstruction by robotics-inspired conformational sampling. *Nature Methods*.

[37] Wang C, Bradley P, Baker D (2007). Protein-protein docking with backbone flexibility. *Journal of Molecular Biology*.

[38] Gront D, Kolinski A (2006). BioShell—a package of tools for structural biology computations. *Bioinformatics*.

[39] Venclovas C, Margelevicius M (2005). Comparative modeling in CASP6 using consensus approach to template selection, sequence-structure alignment, and structure assessment. *Proteins*.

[40] Al-Lazikani B, Sheinerman FB, Honig B (2001). Combining multiple structure and sequence alignments to improve sequence detection and alignment: application to the SH2 domains of Janus kinases. *Proceedings of the National Academy of Sciences of the United States of America*.

